# Suppression of experimental autoimmune encephalomyelitis by interleukin-10 transduced neural stem/progenitor cells

**DOI:** 10.1186/1742-2094-10-117

**Published:** 2013-09-22

**Authors:** Juliane Klose, Nils Ole Schmidt, Arthur Melms, Makoto Dohi, Jun-ichi Miyazaki, Felix Bischof, Bernhard Greve

**Affiliations:** 1 Center of Neurology, Hertie-Institute for Clinical Brain Research, University of Tübingen, Otfried-Müller-Strasse 27, 72076 Tübingen, Germany; 2 Department of Neurosurgery, University Medical Center Hamburg-Eppendorf, Martinistrasse 52, 20246 Hamburg, Germany; 3 Department of Allergy and Rheumatology, Graduate School of Medicine, University of Tokyo, 7-3-1 Hongo, Bunkyo-ku, Tokyo 113-8655, Japan; 4 Division of Stem Cell Regulation Research, Osaka University School of Medicine, 2-2, Yamadagaoka, Suita 565-0871 Osaka, Japan

**Keywords:** Neural stem/progenitor cells, IL-10, EAE

## Abstract

Neural stem/progenitor cells (NSPCs) have the ability to migrate into the central nervous system (CNS) to replace damaged cells. In inflammatory CNS disease, cytokine transduced neural stem cells may be used as vehicles to specifically reduce inflammation and promote cell replacement. In this study, we used NSPCs overexpressing IL-10, an immunomodulatory cytokine, in an animal model for CNS inflammation and multiple sclerosis (MS). Intravenous injection of IL-10 transduced neural stem/progenitor cells (NSPC^IL-10^) suppressed myelin oligodendrocyte glycoprotein aa 35–55 (MOG35-55)- induced experimental autoimmune encephalomyelitis (EAE) and, following intravenous injection, NSPC^IL-10^ migrated to peripheral lymphoid organs and into the CNS. NSPC^IL-10^ suppressed antigen-specific proliferation and proinflammatory cytokine production of lymph node cells obtained from MOG35-55 peptide immunized mice. In this model, IL-10 producing NSPCs act via a peripheral immunosuppressive effect to attenuate EAE.

## Highlights

•Intravenous injected NSPC^IL-10^ suppress EAE.

•NSPC^IL-10^ migrate to lymph nodes, spleen and into the CNS.

•NSPC^IL-10^ inhibit T-cell activation, proliferation and cytokine production.

•This inhibitory effect is independent of indoleamine 2,3-dioxygenase (IDO) and the induction of apoptosis.

## Introduction

Multiple sclerosis (MS) is a disabling inflammatory disease of the central nervous system (CNS) characterized by inflammation, demyelination and neurodegeneration [1]. It is the major disabling disease affecting young individuals. Symptoms such as visual impairment, sensory disturbances and paralysis depend on the location of the lesions in the CNS [2]. Current approved therapies are immunomodulating and immunosuppressive drugs, including IFN-β, natalizumab, fingolimod, glatiramer acetate, mitoxantrone and teriflunomide.

A promising method for a comprehensive therapy of MS integrating various possible therapeutic mechanisms might be the treatment with neural stem/progenitor cells (NSPCs). Early work demonstrated the ability of neural stem cells to proliferate and differentiate into neurons, oligodendrocytes and astrocytes [3-5], and to display an inherent tropism for neuropathology [6]. It has been shown in experimental autoimmune encephalomyelitis (EAE), the animal model of MS, that transplantation of neural stem cells promotes remyelination and reduces brain inflammation [7,8].

NSPCs have been transplanted into recipient animals to replace injured or damaged neural tissue. This has been evaluated in various models of degenerative and inflammatory CNS diseases [9]. Neural stem cells migrate into areas of neuropathology such as malignant glioma [10], brain injury [11] and inflamed CNS tissue during EAE [8,12]. Moreover, neural stem cells have been shown to be able to inhibit inflammation within the CNS in EAE immunized mice [13].

Taking into account this unique pathotropism, we assumed that NSPCs could be used as a cell-based delivery model for immunomodulatory molecules. We choose to use IL-10 as an immunomodulatory cytokine to be delivered by NSPCs into the CNS of mice immunized to develop EAE.

IL-10, a regulatory cytokine mainly produced by T-helper type 2 (Th2) cells, inhibits the activity of Th1 and Th17 cells, the production of proinflammatory cytokines, including TNFα, IL-1 and IFN-γ, and chemokines, as well as the expression of major histocompatibility complex (MHC) class II molecules by antigen-presenting cells (APCs) [14-16]. IL-10 also inhibits macrophages and monocytes, and enhances antibody production and survival of B-lymphocytes [16]. IL-10-deficient mice develop more severe EAE than wild-type mice, overexpression of IL-10 protects mice from EAE [17] and subcutaneous or intranasal treatment reduces disease severity [18]. Thus, IL-10 is an efficient anti-inflammatory cytokine which potently suppresses EAE and is therefore a suitable candidate molecule to be used in our approach. In addition, the immunomodulatory capacity of IFN-β treatment in patients with MS is mediated at least in part by induction of IL-10 production by immune cells.

In this study, we demonstrate that the intravenous injection of IL-10 producing NSPCs in myelin oligodendrocyte glycoprotein aa 35–55 (MOG35-55) peptide immunized C57BL/6 mice during the initial phase of EAE resulted in an attenuated disease course. NSPCs migrated to peripheral lymphoid organs as well as into the CNS, and inhibited proliferation and production of proinflammatory cytokines by autoreactive T-cells. Intravenous injection of IL-10 producing NSPCs attenuates EAE via a peripheral immunosuppressive effect potentially via immunosuppression within the CNS.

## Materials and methods

### Murine NSPCs

NSPCs were established from the frontoparietal brains of 2-week-old C57BL/6 mice and characterized as previously described [19]. NSPC^IL-10^ was established by retroviral transduction with pMSCV-IL10 using a commercially available kit (MSCV Retroviral Expression System, BD Biosciences, San Jose, CA, USA).

The cells were grown as neurospheres in complete NSPC growth medium containing neurobasal medium (Gibco, Karlsruhe, Germany) supplemented with 100 μg/mL penicillin/streptomycin (Gibco), 10 mM L-glutamine (Gibco), 20 μL/mL B-27 supplement (Gibco) and 0.25 μg/mL fungizone (Gibco). Recombinant epidermal growth factor (EGF) (20 ng/mL, PeproTech, London, UK), recombinant basic fibroblast growth factor (FGF) (20 ng/mL, PeproTech) and heparin (1000 IE, Braun, Melsungen, Germany) were added directly to the tissue culture flasks twice a week. NSPCs were routinely harvested and mechanically dissociated into a single cell suspension when the neurospheres reached 200 to 500 mm. Single cell suspensions were used for further passages, intravenous injections and *in vitro* experiments. Supernatants were collected and stored at −80°C for further experiments.

### Animals, EAE induction and NSPC injection

Female C57BL/6 mice were obtained from Charles River (Sulzfeld, Germany) and housed under pathogen-free conditions at the animal facility of the Hertie-Institute for Clinical Brain Research (Tübingen, Germany). All experiments were carried out in accordance with local regulations and approval was obtained in advance (Regierungspräsidium Tübingen, animal experimentation protocol TV N9/04 to BG). At the age of 5 to 6 weeks, mice were immunized with 60 μg MOG35-55, dissolved in 100 μL PBS (PAA Laboratories, Pasching, Austria) and emulsified with 100 μL incomplete Freund’s adjuvant (IFA) (Sigma-Aldrich, Steinheim, Germany) containing 400 μg *Mycobacterium tuberculosis* (Difco Laboratories, Detroit, MI, USA). On the day of immunization and 2 days after immunization, 150 ng *Bordetella pertussis* toxin (Merck, Darmstadt, Germany) was injected intravenously. NSPC^IL-10^, NSPCs or PBS as a negative control was injected intravenously on day 7 post-immunization, or on first sign of disease (1 × 10^6^ cells per injection).

For immunization of 2D2 mice, female 2D2 TCR transgenic mice were obtained from Dr Bettelli [20] and housed under specific pathogen-free conditions. Mice aged 5 to 6 weeks were immunized with 25 μg MOG35-55 dissolved in 100 μL PBS and emulsified with 100 μL IFA containing 400 μg *M tuberculosis*. On day 0 and day 2 pi, 150 ng *B pertussis* toxin was injected intravenously. At day 5 post-immunization, 1 × 10^6^ NSPC^IL10^, NSPCs or PBS was injected intravenously. As a result of the MOG antigen-specific TCR, 2D2 transgenic mice are more sensitive to MOG-specific immunization. Therefore, only a concentration of 25 μg MOG35-55 was used for immunization. At 14 days post-immunization, cells were isolated from draining lymph nodes and cultured in RPMI 1640 medium containing 5 μg/mL or 50 μg/mL MOG35-55 peptide. Proliferation was determined after 72 hours by ^3^H-thymidine incorporation as previously described [21]. Cytokine concentrations in culture supernatants were measured after 48 hours by enzyme-linked immunosorbent assay (ELISA) (eBioscience, San Diego, CA, USA).

Animals were monitored daily starting at least at day 5 post-immunization and clinical signs scored as follows: 0, no paralysis; 1, limp tail; 2, limp tail and weak gait; 3, hind limb paralysis; 4, fore limb paralysis; and 5, death.

### Histology

Prior to injection, NSPCs were labeled with 4 × 10^6^ molar PKH26 dye for 5 minutes at room temperature. Dye reaction was stopped with RPMI 1640 medium containing FBS; cells were washed and injected as previously described. Two weeks after immunization, brain tissue and spinal cord were isolated, fixed with 4% paraformaldehyde (PFA) for 24 hours, incubated for 24 hours in 20 sucrose and frozen in liquid nitrogen. Spleen, lymph nodes, liver and lungs were immediately frozen in liquid nitrogen. Frozen sections were stained with mounting medium containing DAPI (Linaris, Wertheim, Germany) and analyzed for PKH26-labeled cells by fluorescence microscopy. In addition, brain sections were stained with hematoxylin and eosin (H&E), and analyzed by microscopy.

### Spleen cell cultures

Spleens from naive 2D2 TCR transgenic mice and C57BL/6 mice were isolated and cultured with RPMI 1640 medium containing 0.5 μg/mL, 5 μg/mL or 50 μg/mL MOG35-55 peptide, or 0.5 μg/mL or 1 μg/mL concanavalin A (ConA) in the presence of NSPC^IL-10^ or NSPC culture supernatants. To assess effects of NSPC co-cultivation, isolated naive 2D2 or C57BL/6 spleen cells were cultured with NSPC^IL-10^ or NSPCs at a NSPC/spleen cell ratio of 1:1, 1:10 or 1:100 in RPMI 1640 medium containing 5 μg/mL MOG35-55 or 1 μg/ml ConA. Proliferation after 72 hours was detected by a ^3^H-thymidine incorporation assay. Supernatants were collected after 48 hours, and IL-17, IL-2 and IFN-γ concentrations were measured by ELISA. Neurobasal medium served as a control.

### ELISA

Cytokine concentrations were measured by ELISA according to the manufacturer’s instructions (IL-2, IL-10 and IFN-γ, BD Biosciences; IL-17, eBioscience). ELISA plates (NUNC, Kamstrupvej, Denmark) were coated overnight with capture antibody diluted in coating buffer (0.2 M sodium phosphate, pH 6.5). After 1 hour of blocking with assay diluent (PBS containing 10% FBS), cytokine-containing supernatants (eventually diluted with assay diluent) were incubated for 2 hours, followed by 1 hour of incubation with biotinylated secondary antibody and streptavidin-peroxidase. Plates were developed using 3,3’5,5’-tetramethylbenzidine (TMB) (Sigma-Aldrich). Reaction was stopped with 1 M hydrogen chloride and plates were measured at 450 nm using the microplate reader Multiskan Ex (Thermo, Waltham, MA, USA). Standard curves were calculated based on measurements of different concentrations of recombinant cytokines. Plates were washed with PBS containing 0.02% Tween 20 (Roth, Karlsruhe, Germany). All steps were performed at room temperature, except for the coating (4°C).

### ELISPOT

The IL-10 enzyme-linked immunosorbent spot (ELISPOT) was performed using a commercially available kit (Human IL-10 ELISPOT Ready-SET-Go!, eBioscience). Multiscreen TM-HA plates (Millipore, Billerica, MA, USA) were coated overnight with coating antibody diluted in coating buffer. After 1 hour of blocking with RPMI 1640 medium, 4 × 10^5^ NSPC^IL-10^ or NSPCs were incubated at 37°C, 5% CO_2_ for 48 hours. Plates were incubated with biotinylated secondary antibody for 2 hours and subsequently with streptavidin-peroxidase for 45 minutes, and then developed with AEC substrate solution (0.1 M acetate solution, pH 5.0) for 60 minutes. Resulting spots were analyzed with an ELISPOT reader. At every step, the plates were washed with PBS containing 0.02% Tween 20. All steps were performed at room temperature, except for the coating with the capture antibody (4°C).

### RT-PCR

Total RNA was isolated from spleen cells of naive C57BL/6 mice or from cultured NSPC^IL-10^ and NSPCs (using a kit from Seqlab, Göttingen, Germany). RNA was transcribed into cDNA using products from Promega (Madison, WI, USA). PCR reactions were performed in a thermocycler (Biozym, Oldendorf, Germany) using the following conditions: 35 cycles for 1 minute at 94°C, 2 minutes at 55°C and 3 minutes at 72°C. Reaction was completed by incubation at 72°C for 10 minutes. PCR products were separated by gel electrophoresis using 2% agarose gels containing ethidium bromide. PCR primers specific for mouse indoleamine 2,3-dioxygenase (IDO) were 5’GCCCACCGGAACTTCCTTT3’ (forward) and 5’CACTCGTTATAAGCTTTCGTCAAGTC 3’ (reverse). Amplification of β-actin served as a control. PCR primers for mouse β-actin were 5’TGTCCACCTTCCAGCAGATGT 3’ (forward) and 5’AGCTCAGTAACAGTCCGCCTAGA 3’ (reverse) [22].

### IDO inhibition assays

1-methyl-DL-tryptophan (1-MT) (Sigma-Aldrich) was used as an IDO inhibitor. Isolated 2D2 spleen cells were cultured in RPMI 1640 medium containing 5 μg/mL MOG35-55 and NSPC^IL-10^ or NSPCs (NSPC/spleen cell ratio of 1:1) in the presence or absence of 500 μg/mL 1-MT. Proliferation after 72 hours was detected by ^3^H-thymidine incorporation.

### Statistical analysis

EAE experiments with NSPC injection on day 7 after EAE induction were performed with 15 mice per group and EAE experiments with NSPC injection after onset of clinical symptoms with seven mice per group. Proliferation, ELISA and flow cytometry experiments were carried out in triplicate. Values are expressed as mean ± SD. Statistical analysis was performed with SPSS Statistics 17.0 for Windows (IBM Armonk, NY, USA). For the EAE experiments, a survival analysis and analysis of variance (ANOVA) with a post-hoc Tukey-Kramer test were performed. *In vitro* experiments were analyzed using ANOVA with post-hoc Bonferroni testing.

## Results

### NSPC culture

**Figure 1:**
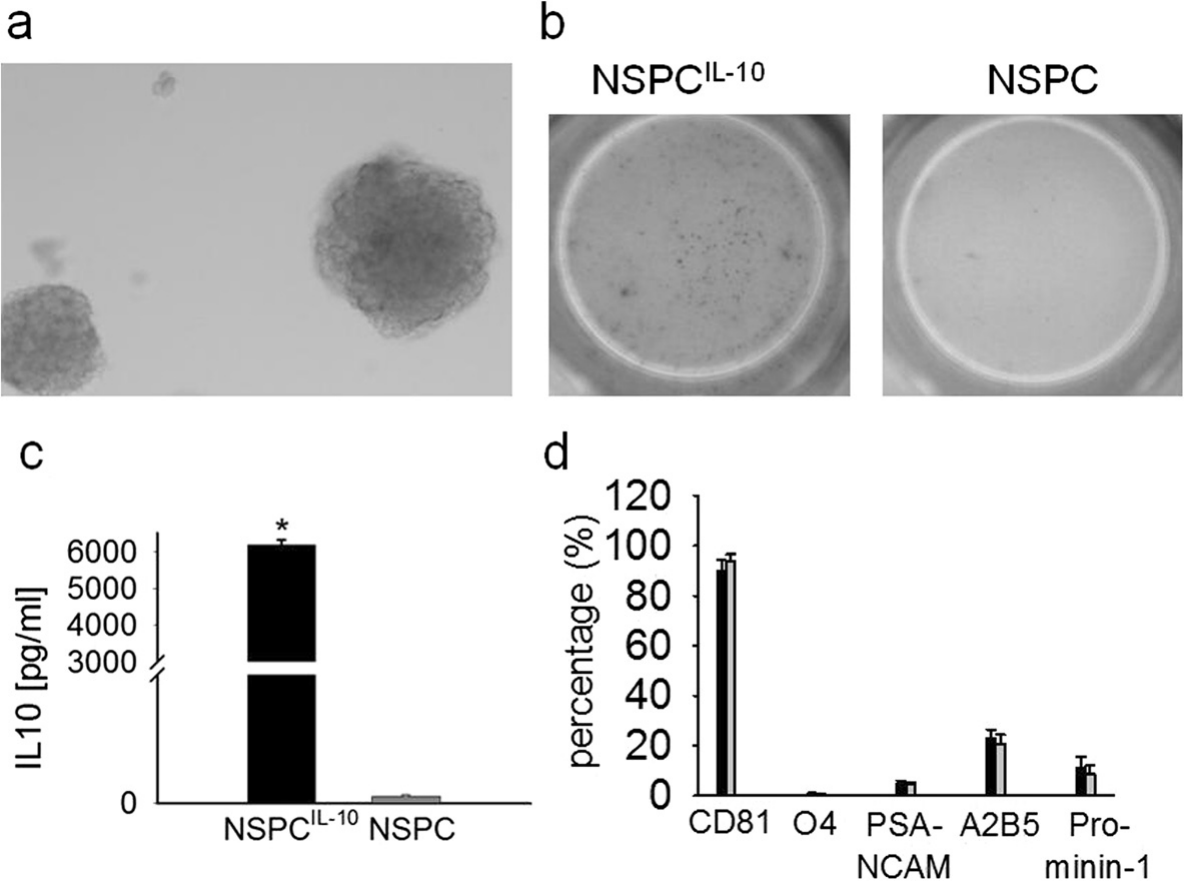
**NSPC and IL-10 production. (a)** Morphology of neurospheres as demonstrated by a phase contrast image of a representative neurosphere culture in presence of 20 ng/mL FGF-2 and 20 ng/mL EGF. **(b)** IL-10 production of NSPC^IL-10^ and NSPCs was determined by IL-10 ELISPOT assay (dots indicating IL-10 producing cells), and **(c)** IL-10 ELISA. *t-test, *P* <0.001. **(d)** Analysis of NSPC^IL-10^ (black bar) and NSPCs (grey bar) for expression of different stem cell markers by flow cytometry. EGF, epidermal growth factor; ELISA, enzyme-linked immunosorbent assay; ELISPOT, enzyme-linked immunosorbent spot; FGF, fibroblast growth factor; IL, interleukin; NSPC, neural stem/progenitor cell; PSA-NCAM, polysialic acid neural cell adhesion molecule.

NSPCs were cultured in the presence of basic FGF and EGF from neurospheres (Figure 1a). As expected, phenotypic characterization by flow cytometry showed multipotency of NSPCs and a percentage of neuronal stem cells among total NSPCs of around 5% (O4^-^ PSA-NCAM^-^ CD81^-^ Prominin-1) [19,23]. IL-10 production of transduced cells was confirmed using an IL-10 ELISPOT assay and an IL-10 ELISA (Figure 1b,c). NSPCs and NSPC^IL-10^ were indistinguishable with regard to expression of differentiation markers (90% expressed CD81, <10% expressed O4 and PSA-NCAM, 10% expressed CD133, and 20% expressed A2B5 (Figure 1d).

### Injected NSPC^IL-10^ suppress EAE when injected during peripheral T-cell activation

**Figure 2:**
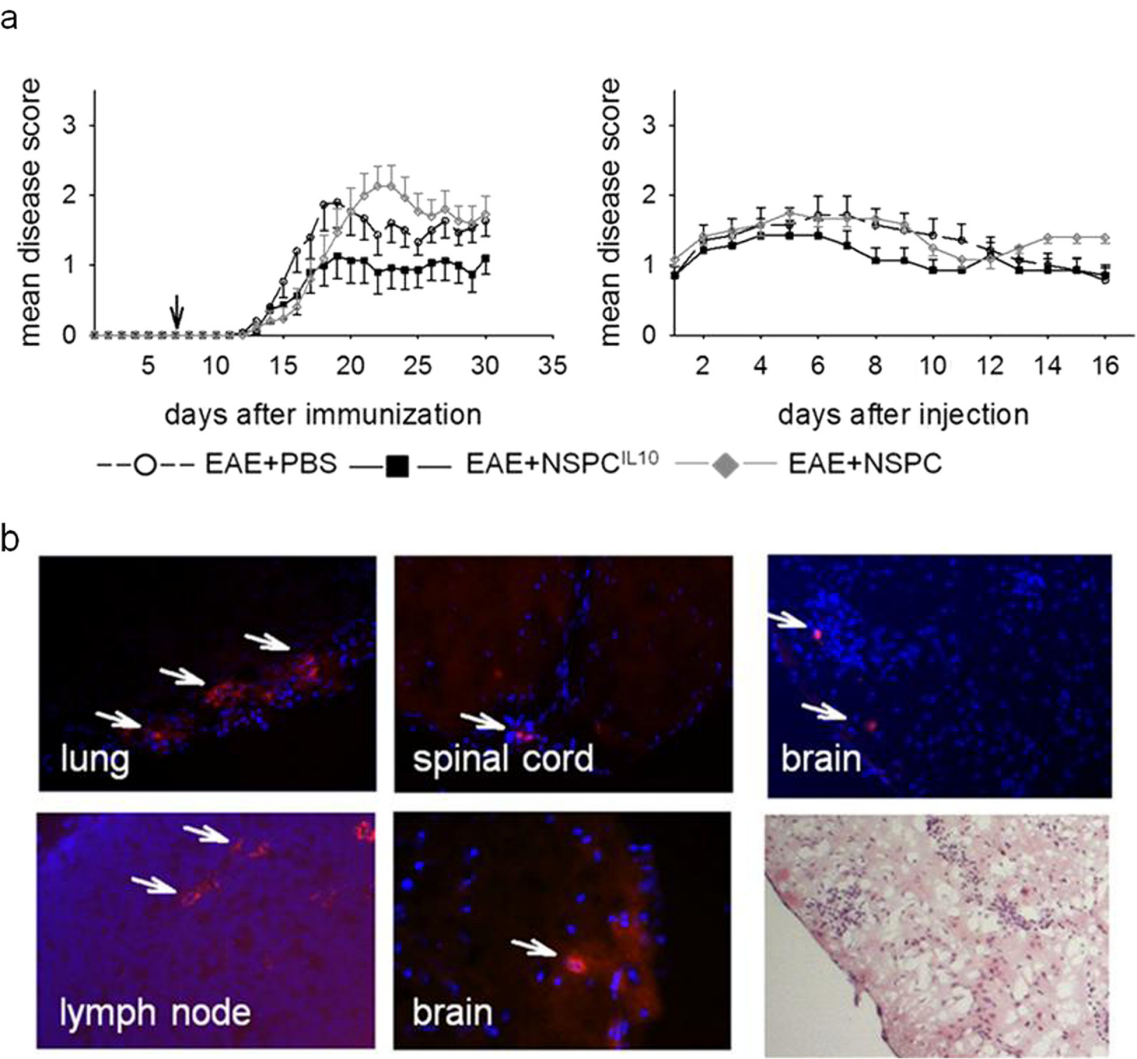
**NSPC**^**IL-10 **^**suppress clinical scores in EAE.** EAE was induced in C57BL/6 mice by subcutaneous injection of 50 μg MOG35-55 peptide in CFA plus intravenous injection of 150 ng *Bordetella pertussis* toxin. **(a)** On day 7 after EAE induction or **(a)** after established EAE disease, mice were intravenously injected with 1 x 10^6^ NSPC^IL-10^, NSPCs or PBS as indicated. **(b)** 1 x 10^6^ PKH26-labeled NSPC^IL-10^, NSPCs or PBS were injected 7 days after induction of EAE in 2D2 mice with MOG35-55. On day 7 after injection, organs were examined by fluorescence microscopy for the presence of PKH26^+^ cells. Cell nuclei were stained with DAPI (blue). Infiltration was analyzed by H&E stain. NSPC^IL-10^ and NSPCs were detected within lungs, spinal cords, lymph nodes and brains of MOG35-55 immunized 2D2 mice and in the CNS within cellular infiltrates. Tissues are shown from NSPC^IL-10^-treated mice as representative examples for NSPC- and NSPC^IL-10^-treated mice. CFA, complete Freund’s adjuvant; CNS, central nervous system; DAPI, 4’,6-diamidino-2-phenylindole; EAE, experimental autoimmune encephalomyelitis; H&E, hematoxylin and eosin; IL, interleukin; MOG35-55, myelin oligodendrocyte glycoprotein aa 35–55; NSPC, neural stem/progenitor cell; PBS, phosphate-buffered saline.

To analyze the effect of NSPC^IL-10^ on the development of CNS-directed autoimmunity, 1 × 10^6^ NSPC^IL-10^ was injected intravenously into C57BL/6 mice on day 7 after EAE induction with MOG35-55 peptide. Disease severity and maximum EAE disease score were reduced in mice that received NSPC^IL-10^ compared to NSPC- or PBS-treated control animals. The mean maximum disease score in NSPC^IL-10^-treated mice was significantly reduced in comparison to NSPC-treated mice or PBS-treated control animals (*P* = 0.02). Furthermore, NSPC^IL-10^-treated mice showed a milder EAE disease score during the whole observation period and the lowest sum score in comparison with control groups (Figure 2a). The effects of NSPC^IL-10^ injection after disease onset were also examined. Injection of 1 × 10^6^ NSPC^IL-10^ into MOG35-55 immunized C57BL/6 mice 1 day after the beginning of clinical EAE only mildly influenced the disease course (Figure 2a).

In order to assess how NSPC^IL-10^ exerts suppressive effects *in vivo*, we determined their migratory properties after intravenous injection into immunized animals. Mice with MOG35-55-induced EAE received intravenous injections of NSPC^IL-10^ and NSPCs labeled with the fluorescent dye PKH26. Two weeks after injection, PKH26^+^ NSPC^IL-10^ and NSPCs were present in lungs, lymph nodes and inflammatory infiltrates within the CNS (Figure 2b), while no PKH26-labeled NSPC^IL-10^ or NSPCs could be detected within the liver.

### NSPC^IL-10^ suppress T-cell proliferation and cytokine production in draining lymph nodes

**Figure 3:**
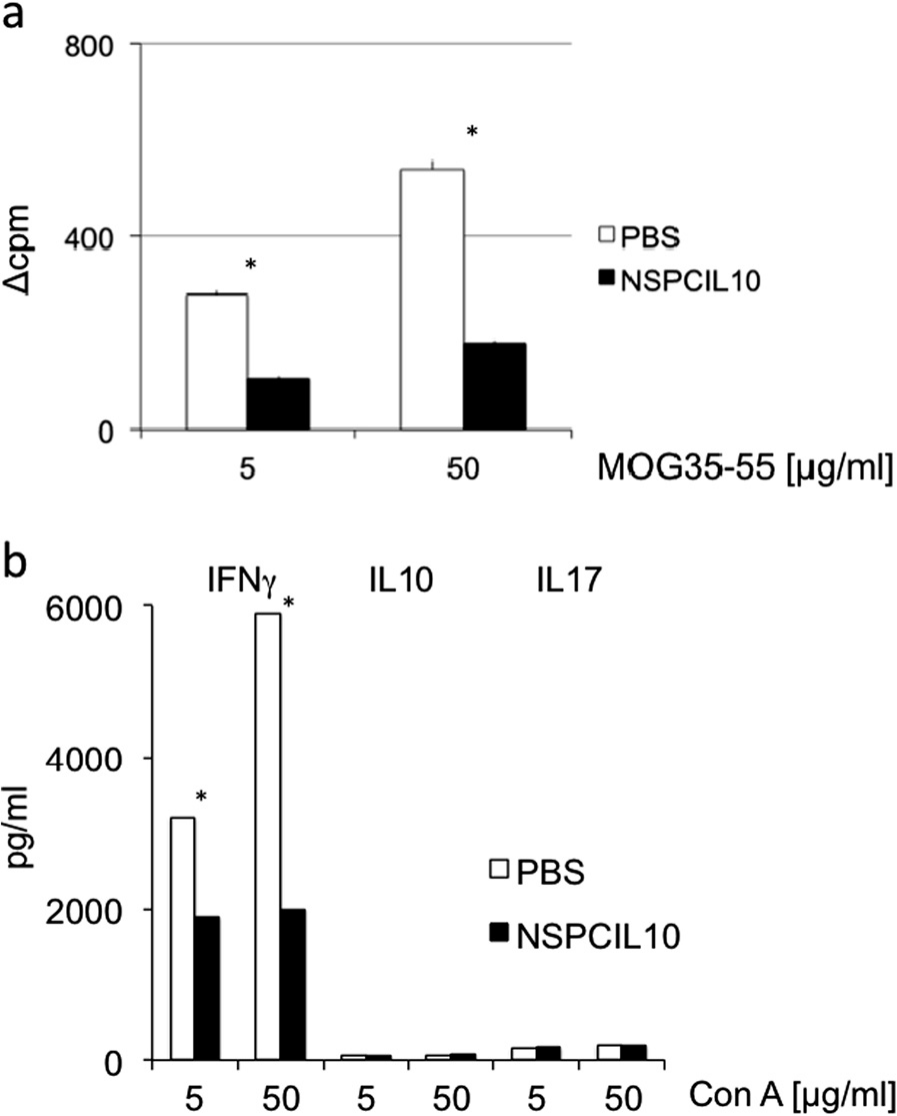
**NSPC**^**IL-10 **^**cells reduce proliferation and IFN-γ production by lymph node cells.** 2D2 mice with MOG35-55-induced EAE received intravenous injections of 1 x 10^6^ NSPC^IL-10^, NSPCs or PBS on day 7 after disease induction. Draining lymph node cells were isolated 7 days after injection and stimulated with MOG35-55 peptide. **(a)** Proliferation was determined by ^3^H-thymidine incorporation, and **(b)** IFN-γ, IL-10 and IL-17 production was determined by ELISA. *P <0.05. NSPC^IL-10^ versus NSPCs are expressed as mean values with SD from three independent experiments. ConA, concanavalin A; EAE, experimental autoimmune encephalomyelitis; ELISA, enzyme-linked immunosorbent assay; IFN, interferon; IL, interleukin; MOG35-55, myelin oligodendrocyte glycoprotein aa 35–55; NSPC, neural stem/progenitor cell; PBS, phosphate-buffered saline.

MOG35-55-induced EAE is a T-cell-mediated disease, and autoreactive T-cells in EAE are activated within peripheral lymph nodes before they migrate into the CNS [24]. Since intravenously injected NSPC^IL-10^ migrate to peripheral lymph nodes, we considered whether NSPC^IL-10^ influence the activity or function of autoreactive T-cells within this compartment. To this end, C57BL/6 mice carrying a transgenic TCR specific for MOG35-55 (2D2 mice) [20] were immunized with MOG35-55 and treated 7 days following immunization with NSPC^IL-10^ or PBS. Draining lymph node cells were isolated 14 days post-immunization, and assessed for proliferation and cytokine production upon stimulation with MOG35-55 peptide. Treatment with NSPC^IL-10^ reduced the proliferative capacity of autoreactive T-cells in draining lymph nodes (Figure 3a) in this transgenic model. In addition, the capacity to produce IFN-γ was reduced in treated animals; IL-17 and IL-10 were only detected at low levels under these experimental conditions (Figure 3b). Transferred NSPC^IL-10^ thus inhibited proliferation and IFN-γ production by autoreactive T-cells at the site of peripheral T-cell activation *in vivo*.

### Immunosuppressive effects of NSPC^IL-10^ and NSPC^IL-10^ supernatant *in vitro*

**Figure 4:**
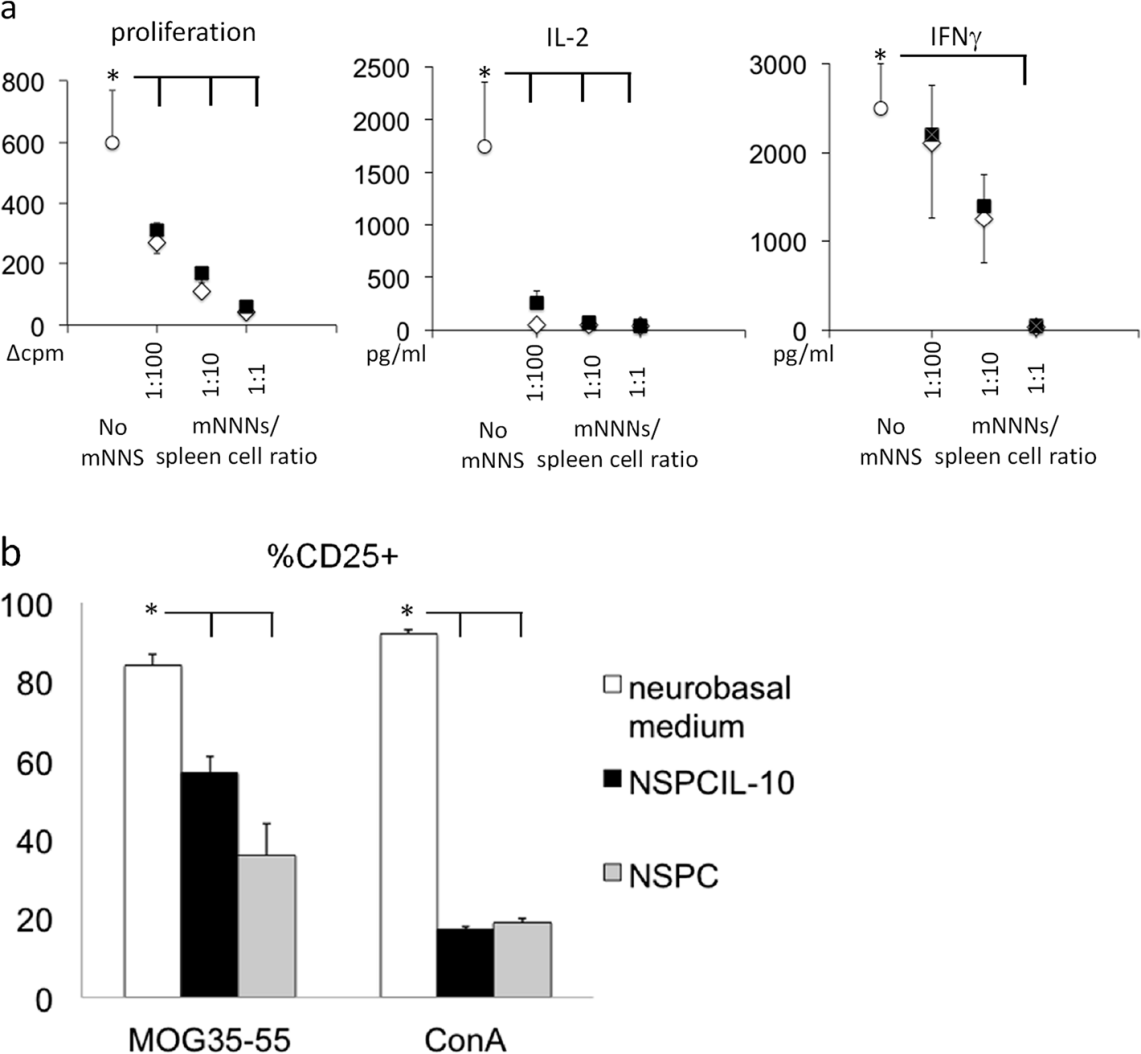
**NSPC**^**IL-10 **^**and NSPCs inhibit proliferation and cytokine production by T-cells. (a)** C57BL/6 spleen cells were polyclonal-activated with ConA in the presence of NSPC^IL-10^ (black squares), NSPCs (open diamonds) or neurobasal medium (open circles) as indicated. Proliferation was determined by ^3^H-thymidine incorporation. IFN-γ and IL-2 production was assessed by ELISA. *P <0.05. Mean values are expressed with SD from three independent experiments. **(b)** Frequency of CD25^+^ cells of CD4 T-cells of MOG35-55-stimulated 2D2 spleen cells or ConA-stimulated spleens cells after cultivation with NSPC^IL-10^, NSPCs or neurobasal medium. Analysis was performed by flow cytometry. *P <0.05. NSPC, neural stem/progenitor cell; ConA, concanavalin A; ELISA enzyme-linked immunosorbent assay; IFN, interferon; IL, interleukin; MOG35-55, myelin oligodendrocyte glycoprotein aa 35–55.

To further investigate the immunosuppressive effects of NSPC^IL-10^, we cultivated NSPC^IL-10^ with MOG35-55-activated spleen cells isolated from naive 2D2 mice at different cell ratios. MOG35-55-activated spleen cells cultivated with NSPCs or neurobasal medium served as controls. NSPC^IL-10^ suppressed proliferation of MOG35-55-activated cells as determined by ^3^H-thymidine incorporation, and production of IL-2 and IFN-γ (Figure 4a). A co-culture ratio of NSPC^IL-10^/spleen cells of 1:1 lead to maximal inhibition of proliferation and cytokine production, and a NSPC^IL-10^/spleen cell ratio of 1:100 still suppressed proliferation and cytokine production.

To assess whether NSPCs and NSPC^IL-10^ influence T-cell activation, we determined the expression of the IL-2 receptor α chain (CD25) on antigen-specific and polyclonal stimulation of spleen cells by flow cytometry. MOG35-55 or ConA-stimulated spleen cells were cultivated in the presence of NSPC^IL-10^, NSPCs or neurobasal medium. NSPCs and NSPC^IL-10^ potently suppressed activation of CD4 T-cells as determined by CD25 expression (Figure 4b).

**Figure 5:**
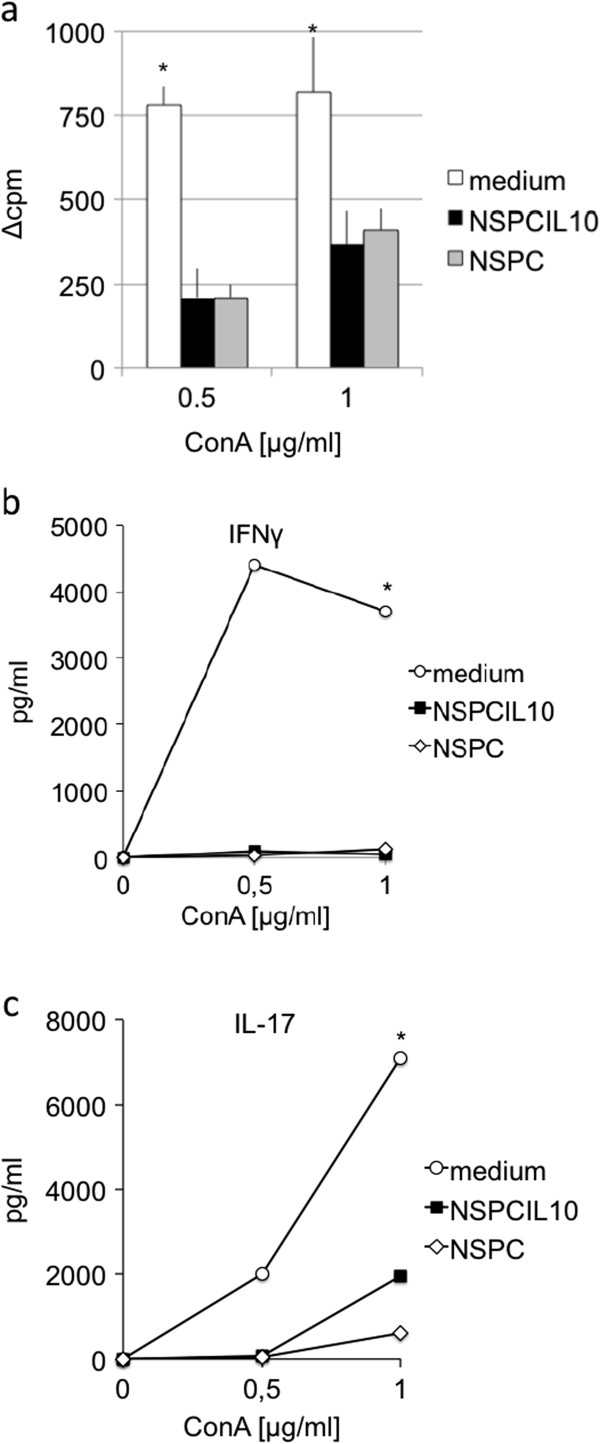
**Supernatant of NSPC**^**IL-10 **^**inhibits proliferation, and the production of IFN-γ and IL-17 by spleen cells.** C57BL/6 spleen cells were stimulated with ConA in the presence of neurobasal medium (white bar, circles) or culture supernatant of NSPC^IL-10^ (black bar, squares) or NSPC (grey bar, diamonds). **(a)** Proliferation was determined by ^3^H-thymidine incorporation, and production of **(b)** IFN-γ and **(c)** IL-17 was assessed by ELISA. *P <0.05. Mean values are expressed with SD from three independent experiments. ConA, concanavalin A; ELISA, enzyme-linked immunosorbent assay; IFN, interferon; IL, interleukin; NSPC, neural stem/progenitor cell.

To evaluate whether immunosuppression by NSPCs and NSPC^IL-10^ is mediated by soluble factors, culture supernatants of NSPCs and NSPC^IL-10^ were collected and incubated with antigen-specific or polyclonal stimulation of spleen cells. Culture supernatants of NSPCs and NSPC^IL-10^ inhibited proliferation, and IFN-γ and IL-17 production of ConA-stimulated cells in comparison to neurobasal medium (Figure 5a,b,c).

### Inhibition of T-cell proliferation by NSPC^IL-10^ is not mediated by IDO or induction of apoptosis

**Figure 6:**
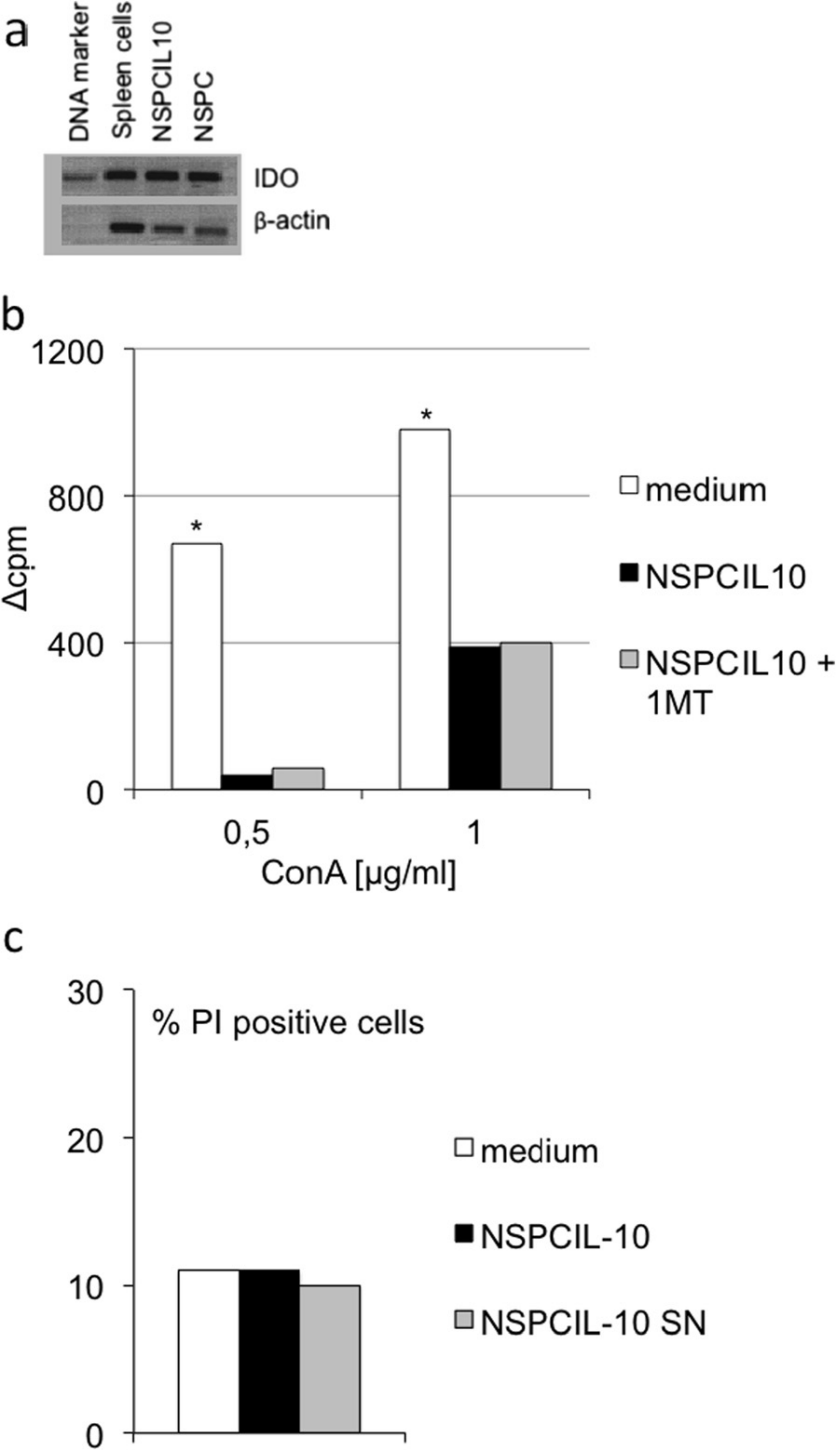
**Immunosuppression of NSPC**^**IL-10 **^**is not mediated by IDO and not due to induction of apoptosis. (a)** NSPC^IL-10^ and NSPCs express IDO. IDO expression was detected by RT-PCR in spleens, NSPC^IL-10^ and NSPCs. **(b)** Inhibition of IDO by 1-MT does not abrogate the immunosuppression mediated by co-culture of NSPC^IL-10^. C57BL/6 spleen cells were activated with ConA in the presence of neurobasal medium, NSPC^IL-10^ or NSPC^IL-10^ and 1-MT. Proliferation was determined by ^3^H-thymidine incorporation. **(c)** NSPC^IL-10^ and NSPC^IL-10^ culture supernatants do not induce apoptosis in activated spleen cells. C57BL/6 spleen cells were stimulated with ConA in the presence of medium, NSPC^IL-10^ and NSPC^IL-10^ culture supernatant. After 24 hours, the frequency of PI^+^ cells was determined by flow cytometry. 1-MT, 1-methyl-DL-tryptophan; ConA, concanavalin A; IDO, indoleamine 2,3-dioxygenase; IL, interleukin; NSPC, neural stem/progenitor cell; PI, propidium iodide; RT-PCR, reverse transcription polymerase chain reaction.

NSPC^IL-10^ inhibited T-cell proliferation. T-cell proliferation depends on the availability of tryptophan, which is closely regulated by the tryptophan-degrading enzyme IDO [25]. IDO expression is induced in many cell types, including macrophages, dendritic cells, fibroblasts, microglia and astrocytes by the proinflammatory cytokines IFN-γ and IFN-β. We considered whether immunosuppression by NSPC^IL-10^ is related to IDO activity. *In vitro* cultivated NSPC^IL-10^ and NSPCs expressed large amounts of IDO as demonstrated by RT-PCR (Figure 6a). Inhibition of IDO by the tryptophan analogue 1-MT [26] did not alter the ability of NSPC^IL-10^ to inhibit polyclonal T-cell proliferation, indicating that immunosuppression by NSPC^IL-10^ is not mediated by IDO (Figure 6b).

We also examined whether NSPC^IL-10^ mediated immunosuppression by induction of apoptotic cell death. We found no enhanced induction of apoptotic cell death in polyclonal stimulation of spleen cells after cultivation with NSPC^IL-10^ or NSPC^IL-10^ culture supernatants (Figure 6c). We conclude that the immunosuppressive effects of NSPC^IL-10^ are not due to enhanced induction of apoptosis in CNS-specific or polyclonal T-cells.

## Discussion

Different sources of somatic stem cells, including neural progenitor cells [8,27], hematopoietic [28,29] and mesenchymal stem cells [30-32], are currently explored for their immunomodulatory capacity in trauma, stroke, neurodegenerative diseases and CNS-directed autoimmunity. NSPCs have the unique ability to migrate directly into the damaged brain [6,27,33,34], and cytokines released by activated cells during the disease process act as chemoattractants for NSPCs [11,35-37]. We made use of the specific tropism of neural stem cells to deliver IL-10 into sites of inflammation and neurodegeneration in an animal model of CNS inflammation. To this end, we injected IL-10 producing NSPCs in MOG35-55 peptide immunized mice and observed EAE disease severity, migration pattern and immune reactions to evaluate the therapeutic potential of IL-10 producing NSPCs.

Injection of NSPC^IL-10^ at the time of peripheral T-cell activation induced a robust reduction of disease severity, while injection after onset of clinical symptoms did not significantly alter the disease course. After intravenous injection, NSPCs and NSPC^IL-10^ migrated into the CNS, as well as into peripheral lymph nodes and spleen. These results indicate that NSPCs are suitable vectors for drug delivery into the diseased CNS but also migrate to peripheral lymphoid organs. This is consistent with earlier studies using neural precursor cells [38].

We analyzed the immune reaction defined by proliferation and cytokine expression of lymph node cells from MOG35-55 immunized 2D2 mice treated with NSPC^IL-10^. NSPC^IL-10^ suppressed *ex vivo* proliferation of T-cells isolated during the peak of the disease. Moreover, NSPC^IL-10^ suppressed production of IFN-γ but not IL-10 by MOG35-55-specific T-cells, indicating suppression of Th1-related cytokines. During the induction phase of EAE, encephalitogenic T-cells migrate from draining lymph nodes to the spleen and subsequently into the CNS [39]. Collectively, these results indicate that intravenously injected NSPC^IL-10^ suppress EAE by migrating into peripheral lymphoid organs where they inhibit T-cell proliferation and cytokine production.

This immunosuppressive effect of NSPC^IL-10^ on immune cells was confirmed *in vitro*. NSPC^IL-10^ suppressed proliferation and IL-2 and IFN-γ production by antigen-specific and polyclonal stimulation of spleen cells, and the presence of NSPC^IL-10^ cells also inhibited expression of IL2Rα demonstrating reduced T-cell activation. This inhibitory effect is mediated at least in part by direct interaction of NSPC^IL-10^ and T-cells as it was also observed after mitogen activation of T-cells in the absence of APCs. In addition, IL-10 could reduce inflammation by reducing MHC class II expression by APCs.

Other studies have shown similar peripheral immunosuppressive effects after peripheral injection of neural stem cells or neural precursor cells. In these studies, intravenously injected neural precursor cells migrated to peripheral lymphoid organs and inhibited proliferation, activation or release of proinflammatory cytokines by autoreactive T-cells [7]. Subcutaneously injected neural stem cells migrated into lymph nodes and inhibited the generation of effector T-cells [38]. In our experiments, disease suppression was maximal when NSPC^IL-10^ was injected at the time of peripheral T-cell activation. This observation further supports the notion that NSPC^IL-10^ attenuates EAE by inhibiting T-cell activation in peripheral lymphoid organs rather than within the CNS.

Non-transfected NSPCs were used as a control in all experiments. Injection of NSPCs at the time of peripheral T-cell activation did not result in reduction of clinical EAE as opposed to injection of NSPC^IL-10^. Other studies have previously shown beneficial effects of neural stem cells or neural precursor cells on EAE disease course [7,27,33,38]. Differences in EAE models, mouse-strains, NSPC-types or time points of injection could contribute to these different results. In this study, the migration pattern of NSPCs was similar compared to the pattern of NSPC^IL-10^ and also comparable to previous observations. We detected NSPCs in peripheral lymphoid organs and within the CNS after intravenous injection similar to our results with NSPC^IL-10^. We also found an inhibitory effect on proliferation and IL-2/IFN-γ production of antigen-specific activated lymph node cells and spleen cells isolated after 35 days pi, and during the disease peak. Moreover, NSPCs inhibited proliferation and IL-2/IFN-γ production of antigen-specific and polyclonal stimulation of spleen cells in co-cultivation experiments. Thus, we were able to replicate *in vitro* effects of neural stem cells or NSPCs described previously. However, in contrast to previous studies, we were not able to show *in vivo* effects of non-transfected NSPCs. In general, the inhibitory *in vitro* effects of NSPCs were weaker than those of NSPC^IL-10^.

Another possible mechanism by which NSPCs may inhibit immune cells could be the induction of apoptosis. Previous studies have shown that mesenchymal stem cells [40] and neural multipotent precursor cells [8] induced apoptosis in activated T-cells. In addition, Yang *et al*. [13] demonstrated that IL-10-expressing neural stem cells induce apoptosis in CD4^+^ T-cells. In contrast, in our experiments, NSPC^IL-10^ or culture supernatants of NSPC^IL-10^ did not induce apoptosis of antigen-specific or polyclonal stimulation of spleen cells. We further investigated if IDO expression could be responsible for the inhibitory effects of NSPC^IL-10^. IDO is the first and rate-limiting enzyme in the degradation of tryptophan to kynurenine [41]. In healthy individuals, IDO is expressed only at low levels but expression increases after infection or inflammation [25]. It was shown that in proteolipid protein (PLP)-induced EAE, IDO induction leads to reduced neuroinflammation [42] and that mesenchymal stem cells suppress proliferation of T-cells via expression of IDO [43]. NSPC^IL-10^ and NSPCs express IDO in culture as demonstrated by RT-PCR, but there was no association between IDO expression and inhibition of proliferation mediated by NSPC^IL-10^.

IL-10 is an anti-inflammatory cytokine mainly produced by Th2 cells, and inhibits the activity of Th1 lymphocytes, natural killer (NK) cells and macrophages [16]. The inhibitory effects of IL-10 may be mediated by inhibition of the co-stimulatory signals CD80 and CD86 on APCs [44], or by induction of immunosuppressive regulatory T-cells (Tregs), such as Tr1, which in turn inhibit cell proliferation through production of IL-10 or transforming growth factor (TGF)-β [45]. In addition, it has previously been shown that stimulation of CD4^+^ T-cells in the presence of IL-10 induces a state of unresponsiveness with reduced proliferation and IL-2 production [46]. Our co-cultivation experiments using NSPC^IL-10^ showed that there was no decreased expression of CD80 or CD86 on antigen-specific stimulated spleen cells or a higher percentage of CD4^+^ FoxP3^+^ cells (data not shown), but we found reduced expression of IL2Rα, reduced IL-2 production and proliferation when spleen cells were stimulated in the presence of NSPC^IL-10^. Therefore, IL-10 producing NSPCs seem to mediate their effects by inducing a non-responsive state in T-cells. In a recent study, Yang *et al*. [13] evaluated the effects of IL-10 producing neural stem cells on EAE. After intravenous injection, cells were detected in peripheral lymphoid organs and were shown to act via an immunosuppressive mechanism which was enhanced through IL-10 production. In contrast to our study, these experiments also showed a positive effect when cells were injected after onset of clinical EAE.

As proof of concept, we have shown that IL-10 producing NSPCs ameliorate the clinical disease course of EAE. NSPCs may be used as vehicles to transport immunomodulatory molecules to sites of T-cell activation and inflammation in order to attenuate autoimmune processes. These results, however, do not rule out immunomodulatory functions of NSPC^IL-10^ within the brain and further work is needed to explore this. The finding that NSPC^IL-10^ only marginally suppresses EAE disease severity when administered after EAE symptoms has been established and indicates that the time of administration of NSPC^IL-10^ critically determines its potency to suppress CNS autoimmunity. Whether such an approach would be feasible in patients with MS remains to be established.

## Abbreviations

1-MT: 1-methyl-DL-tryptophan; AEC: 3-amino-9-ethylcarbazole; ANOVA: Analysis of variance; APC: Antigen-presenting cell; CFA: Complete Freund’s adjuvant; CNS: Central nervous system; ConA: Concanavalin A; DAPI: 4’,6-diamidino-2-phenylindole; EAE: Experimental autoimmune encephalomyelitis; EGF: Epidermal growth factor; ELISA: Enzyme-linked immunosorbent assay; ELISPOT: Enzyme-linked immunosorbent spot; FBS: Fetal bovine serum; FGF: Fibroblast growth factor; H&E: Hematoxylin and eosin; IDO: Indoleamine 2,3-dioxygenase; IFA: Incomplete Freund’s adjuvant; IFN: Interferon; IL: Interleukin; MHC: Major histocompatibility complex; MOG: Myelin oligodendrocyte glycoprotein; MOG35-55: Myelin oligodendrocyte glycoprotein aa 35–55; MS: Multiple sclerosis; MSCV: Murine stem cell virus; NK: Natural killer; NSPC: Neural stem/progenitor cell; PBS: Phosphate-buffered saline; PFA: Paraformaldehyde; PI: Propidium iodide; PLP: Proteolipid protein; PSA-NCAM: Polysialic acid neural cell adhesion molecule; RPMI: Roswell Park Memorial Institute; RT-PCR: Reverse transcription polymerase chain reaction; TCR: T-cell receptor; TGF: Transforming growth factor; Th: T-helper; TMB: 3,3’5,5’-tetramethylbenzidine; TNF: Tumor necrosis factor; Treg: Regulatory T-cell.

## Competing interests

The authors declare that they have no competing interests.

## Authors’ contributions

JK, BG and AM designed the experiments. JK performed experiments, JK and BG analyzed the data and performed statistical analysis. JK, BG and FB interpreted the data, wrote the manuscript and prepared the graphics. NOS, MD and JM provided critical materials. All authors read and approved the final version of the manuscript.

## References

[B1] Frohman EM Racke MK Raine CS Multiple sclerosis–the plaque and its pathogenesis N Engl J Med 2006 354 942 955 10.1056/NEJMra052130 16510748

[B2] Rejdak K Jackson S Giovannoni G Multiple sclerosis: a practical overview for clinicians Br Med Bull 2010 95 79 104 10.1093/bmb/ldq017 20603280

[B3] Deierborg T Roybon L Inacio AR Pesic J Brundin P Brain injury activates microglia that induce neural stem cell proliferation ex vivo and promote differentiation of neurosphere-derived cells into neurons and oligodendrocytes Neuroscience 2010 171 1386 1396 10.1016/j.neuroscience.2010.09.045 20883748

[B4] Picard-Riera N Decker L Delarasse C Goude K Nait-Oumesmar B Liblau R Pham-Dinh D Evercooren AB Experimental autoimmune encephalomyelitis mobilizes neural progenitors from the subventricular zone to undergo oligodendrogenesis in adult mice Proc Natl Acad Sci USA 2002 99 13211 13216 10.1073/pnas.192314199 12235363 PMC130612

[B5] Reynolds BA Tetzlaff W Weiss S A multipotent EGF-responsive striatal embryonic progenitor cell produces neurons and astrocytes J Neurosci 1992 12 4565 4574 1432110 10.1523/JNEUROSCI.12-11-04565.1992 PMC6575989

[B6] Müller FJ Snyder EY Loring JF Gene therapy: can neural stem cells deliver? Nat Rev Neurosci 2006 7 75 84 16371952 10.1038/nrn1829

[B7] Einstein O Fainstein N Vaknin I Mizrachi-Kol R Reihartz E Grigoriadis N Lavon I Baniyash M Lassmann H Ben-Hur T Neural precursors attenuate autoimmune encephalomyelitis by peripheral immunosuppression Ann Neurol 2007 61 209 218 10.1002/ana.21033 17187374

[B8] Pluchino S Zanotti L Rossi B Brambilla E Ottoboni L Salani G Martinello M Cattalini A Bergami A Furlan R Comi G Constantin G Martino G Neurosphere-derived multipotent precursors promote neuroprotection by an immunomodulatory mechanism Nature 2005 436 266 271 10.1038/nature03889 16015332

[B9] Martino G Pluchino S The therapeutic potential of neural stem cells Nat Rev Neurosci 2006 7 395 406 16760919 10.1038/nrn1908

[B10] Aboody KS Brown A Rainov NG Bower KA Liu S Yang W Small JE Herrlinger U Ourednik V Black PM Breakefield XO Snyder EY Neural stem cells display extensive tropism for pathology in adult brain: evidence from intracranial gliomas Proc Natl Acad Sci USA 2000 97 12846 12851 10.1073/pnas.97.23.12846 11070094 PMC18852

[B11] Sun L Lee J Fine HA Neuronally expressed stem cell factor induces neural stem cell migration to areas of brain injury J Clin Invest 2004 113 1364 1374 15124028 10.1172/JCI20001 PMC398428

[B12] Chu K Kim M Jeong SW Kim SU Yoon BW Human neural stem cells can migrate, differentiate, and integrate after intravenous transplantation in adult rats with transient forebrain ischemia Neurosci Lett 2003 343 129 133 10.1016/S0304-3940(03)00174-5 12759181

[B13] Yang J Jiang Z Fitzgerald DC Ma C Yu S Li H Zhao Z Li Y Ciric B Curtis M Adult neural stem cells expressing IL-10 confer potent immunomodulation and remyelination in experimental autoimmune encephalitis J Clin Invest 2009 119 3678 3691 10.1172/JCI37914 19884657 PMC2786785

[B14] Asadullah K Sterry W Volk HD Interleukin-10 therapy–review of a new approach Pharmacol Rev 2003 55 241 269 10.1124/pr.55.2.4 12773629

[B15] Ding L Linsley PS Huang LY Germain RN Shevach EM IL-10 inhibits macrophage costimulatory activity by selectively inhibiting the up-regulation of B7 expression J Immunol 1993 151 1224 1234 7687627

[B16] Moore KW De-Waal MR Coffman RL O’Garra A Interleukin-10 and the interleukin-10 receptor Annu Rev Immunol 2001 19 683 765 10.1146/annurev.immunol.19.1.683 11244051

[B17] Bettelli E Das MP Howard ED Weiner HL Sobel RA Kuchroo VK IL-10 is critical in the regulation of autoimmune encephalomyelitis as demonstrated by studies of IL-10- and IL-4-deficient and transgenic mice J Immunol 1998 161 3299 3306 9759845

[B18] Xiao BG Bai XF Zhang GX Link H Suppression of acute and protracted-relapsing experimental allergic encephalomyelitis by nasal administration of low-dose IL-10 in rats J Neuroimmunol 1998 84 230 237 10.1016/S0165-5728(97)00264-6 9628468

[B19] Hansen K Müller FJ Messing M Zeigler F Loring JF Lamszus K Westphal M Schmidt NO A 3-dimensional extracellular matrix as a delivery system for the transplantation of glioma-targeting neural stem/progenitor cells Neuro Oncol 2010 12 645 654 10.1093/neuonc/noq002 20156807 PMC2940655

[B20] Bettelli E Pagany M Weiner HL Linington C Sobel RA Kuchroo VK Myelin oligodendrocyte glycoprotein-specific T cell receptor transgenic mice develop spontaneous autoimmune optic neuritis J Exp Med 2003 197 1073 1081 10.1084/jem.20021603 12732654 PMC2193967

[B21] Greve B Weissert R Hamdi N Bettelli E Sobel RA Coyle A Kuchroo VK Rajewsky K Schmidt-Supprian M I kappa B kinase 2/beta deficiency controls expansion of autoreactive T cells and suppresses experimental autoimmune encephalomyelitis J Immunol 2007 179 179 185 17579036 10.4049/jimmunol.179.1.179

[B22] Sheng H Wang Y Jin Y Zhang Q Zhang Y Wang L Shen B Yin S Liu W Cui L Li N A critical role of IFNgamma in priming MSC-mediated suppression of T cell proliferation through up-regulation of B7-H1 Cell Res 2008 18 846 857 10.1038/cr.2008.80 18607390

[B23] Galli R Gritti A Bonfanti L Vescovi AL Neural stem cells: an overview Circ Res 2003 92 598 608 10.1161/01.RES.0000065580.02404.F4 12676811

[B24] Bischof F Hofmann M Schumacher TN Vyth-Dreese FA Weissert R Schild H Kruisbeek AM Melms A Analysis of autoreactive CD4 T cells in experimental autoimmune encephalomyelitis after primary and secondary challenge using MHC class II tetramers J Immunol 2004 172 2878 2884 14978089 10.4049/jimmunol.172.5.2878

[B25] Mellor AL Munn DH IDO expression by dendritic cells: tolerance and tryptophan catabolism Nat Rev Immunol 2004 4 762 774 10.1038/nri1457 15459668

[B26] Mellor AL Munn DH Tryptophan catabolism and T-cell tolerance: immunosuppression by starvation? Immunol Today 1999 20 469 473 10.1016/S0167-5699(99)01520-0 10500295

[B27] Einstein O Karussis D Grigoriadis N Mizrachi-Kol R Reinhartz E Abramsky O Ben-Hur T Intraventricular transplantation of neural precursor cell spheres attenuates acute experimental allergic encephalomyelitis Mol Cell Neurosci 2003 24 1074 1082 10.1016/j.mcn.2003.08.009 14697670

[B28] Parr AM Tator CH Keating A Bone marrow-derived mesenchymal stromal cells for the repair of central nervous system injury Bone Marrow Transplant 2007 40 609 619 10.1038/sj.bmt.1705757 17603514

[B29] Van-Wijmeersch B Sprangers B Rutgeerts O Lenaerts C Landuyt W Waer M Billiau AD Dubois B Allogeneic bone marrow transplantation in models of experimental autoimmune encephalomyelitis: evidence for a graft-versus-autoimmunity effect Biol Blood Marrow Transplant 2007 13 627 637 10.1016/j.bbmt.2007.03.001 17531772

[B30] Kassis I Grigoriadis N Gowda-Kurkalli B Mizrachi-Kol R Ben-Hur T Slavin S Abramsky O Karussis D Neuroprotection and immunomodulation with mesenchymal stem cells in chronic experimental autoimmune encephalomyelitis Arch Neurol 2008 65 753 761 10.1001/archneur.65.6.753 18541795

[B31] Li Y Chen J Chen XG Wang L Gautam SC Xu YX Katakowski M Zhang LJ Lu M Janakiraman N Chopp M Human marrow stromal cell therapy for stroke in rat: neurotrophins and functional recovery Neurology 2002 59 514 523 10.1212/WNL.59.4.514 12196642

[B32] Park HJ Lee PH Bang OY Lee G Ahn YH Mesenchymal stem cells therapy exerts neuroprotection in a progressive animal model of Parkinson’s disease J Neurochem 2008 107 141 151 10.1111/j.1471-4159.2008.05589.x 18665911

[B33] Ben-Hur T Ben-Menachem O Furer V Einstein O Mizrachi-Kol R Grigoriadis N Effects of proinflammatory cytokines on the growth, fate, and motility of multipotential neural precursor cells Mol Cell Neurosci 2003 24 623 631 10.1016/S1044-7431(03)00218-5 14664813

[B34] Pluchino S Quattrini A Brambilla E Gritti A Salani G Dina G Galli R Del-Carro U Amadio S Bergami A Furlan R Comi G Vescovi AL Martino G Injection of adult neurospheres induces recovery in a chronic model of multiple sclerosis Nature 2003 422 688 694 10.1038/nature01552 12700753

[B35] Schmidt NO Przylecki W Yang W Ziu M Teng Y Kim SU Black PM Aboody KS Carroll RS Brain tumor tropism of transplanted human neural stem cells is induced by vascular endothelial growth factor Neoplasia 2005 7 623 629 10.1593/neo.04781 16036113 PMC1501284

[B36] Schmidt NO Koeder D Messing M Mueller FJ Aboody KS Kim SU Black PM Carroll RS Westphal M Lamszus K Vascular endothelial growth factor-stimulated cerebral microvascular endothelial cells mediate the recruitment of neural stem cells to the neurovascular niche Brain Res 2009 1268 24 37 19285048 10.1016/j.brainres.2009.02.065

[B37] Seabrook TJ Littlewood-Evans A Brinkmann V Pöllinger B Schnell C Hiestand PC Angiogenesis is present in experimental autoimmune encephalomyelitis and pro-angiogenic factors are increased in multiple sclerosis lesions J Neuroinflammation 2010 7 95 10.1186/1742-2094-7-95 21176212 PMC3022818

[B38] Pluchino S Gritti A Blezer E Amadio S Brambilla E Borsellino G Cossetti C Del-Carro U Comi G t Hart B Vescovi A Martino G Human neural stem cells ameliorate autoimmune encephalomyelitis in non-human primates Ann Neurol 2009 66 343 354 10.1002/ana.21745 19798728

[B39] Flugel A Berkowicz T Ritter T Labeur M Jenne DE Li Z Ellwart JW Willem M Lassmann H Wekerle H Migratory activity and functional changes of green fluorescent effector cells before and during experimental autoimmune encephalomyelitis Immunity 2001 14 547 560 10.1016/S1074-7613(01)00143-1 11371357

[B40] Plumas J Chaperot L Richard MJ Molens JP Bensa JC Favrot MC Mesenchymal stem cells induce apoptosis of activated T cells Leukemia 2005 19 1597 1604 10.1038/sj.leu.2403871 16049516

[B41] Takikawa O Yoshida R Kido R Hayaishi O Tryptophan degradation in mice initiated by indoleamine 2,3-dioxygenase J Biol Chem 1986 261 3648 3653 2419335

[B42] Kwidzinski E Bunse J Aktas O Richter D Mutlu L Zipp F Nitsch R Bechmann I Indolamine 2,3-dioxygenase is expressed in the CNS and down-regulates autoimmune inflammation FASEB J 2005 19 1347 1349 15939737 10.1096/fj.04-3228fje

[B43] Shi Y Hu G Su J Li W Chen Q Shou P Xu C Chen X Huang Y Zhu Z Huang X Han X Xie N Ren G Mesenchymal stem cells: a new strategy for immunosuppression and tissue repair Cell Res 2010 20 510 518 10.1038/cr.2010.44 20368733

[B44] De-Waal MR Haanen J Spits H Roncarolo MG Te-Velde A Figdor C Johnson K Kastelein R Yssel H De-Vries JE Interleukin 10 (IL-10) and viral IL-10 strongly reduce antigen-specific human T cell proliferation by diminishing the antigen-presenting capacity of monocytes via downregulation of class II major histocompatibility complex expression J Exp Med 1991 174 915 924 10.1084/jem.174.4.915 1655948 PMC2118975

[B45] Vignali DA Collison LW Workman CJ How regulatory T cells work Nat Rev Immunol 2008 8 523 532 10.1038/nri2343 18566595 PMC2665249

[B46] Groux H Bigler M De-Vries JE Roncarolo MG Inhibitory and stimulatory effects of IL-10 on human CD8+ T cells J Immunol 1998 160 3188 3193 9531274

